# Impact of COVID-19 on patients with rheumatic complications of cancer immunotherapy: results of a registry survey

**DOI:** 10.1136/jitc-2020-001550

**Published:** 2020-10-16

**Authors:** Nilasha Ghosh, Aidan Tirpack, Karmela K Chan, Anne R Bass

**Affiliations:** 1 Hospital for Special Surgery Division of Rheumatology, New York, New York, USA; 2 Weill Cornell Medicine, New York, New York, USA

**Keywords:** immunotherapy, autoimmunity, programmed cell death 1 receptor

## Abstract

Immune checkpoint inhibitors (ICI) block negative regulatory molecules, such as CTLA-4, PD-1 and PD-L1, in order to mount an antitumor response. T cells are important for antiviral defense, but it is not known whether patients with cancer treated with ICI are more or less vulnerable to viral infections such as COVID-19. Furthermore, immunosuppressive treatment of immune-related adverse events (irAE) may also impact infection risk. Rheumatic irAEs are often persistent, and can require long-term treatment with immunosuppressive agents. The aim of this study was to determine the incidence of COVID-19 infection and assess changes in ICI and immunosuppressive medication use among patients enrolled in a prospective rheumatic irAE registry during the height of the COVID-19 pandemic. On April 16 2020, following the ‘surge’ of COVID-19 infections in the New York Tri-State area, we sent a 23-question survey to 88 living patients enrolled in a single institutional registry of patients with rheumatic irAE. Questions addressed current cancer and rheumatic irAE status, ICI and immunosuppressant medication use, history of COVID-19 symptoms and/or diagnosed infection. A follow-up survey was sent out 6 weeks later. Sixty-five (74%) patients completed the survey. Mean age was 63 years, 59% were female, 70% had received anti-PD-(L)1 monotherapy and 80% had had an irAE affecting their joints. Six patients (10%) had definite or probable COVID-19, but all recovered uneventfully, including two still on ICI and on low-to-moderate dose prednisone. Of the 25 on ICI within the last 6 months, seven (28%) had their ICI held due to the pandemic. In patients on immunosuppression for irAE, none had changes made to those medications as a result of the pandemic. The incidence of COVID-19 was no higher in patients still on ICI. Ten percent of rheumatic irAE patients developed COVID-19 during the NY Tri-state ‘surge’ of March–April 2020. Oncologists held ICI in a quarter of the patients still on them, particularly women, those on anti-PD-(L)1 monotherapy, and those who had had a good cancer response. The incidence of COVID-19 was no higher on patients still on ICI. None of the patients on disease-modifying antirheumatic drugs or biological immunosuppressive medications developed COVID-19.

Immune checkpoint inhibitors (ICI) block negative regulatory molecules, such as CTLA-4, PD-1 and PD-L1, to enhance a T-cell-mediated antitumor response. Through the upregulation of these T-cells and subsequent inflammatory response, ICI also cause immune-related adverse events (irAE) that can target multiple tissues.^1^ ICI-induced rheumatic irAE, such as inflammatory arthritis, can persist for months to years, even after ICI has been discontinued. Treating the arthritis may require long-term use of immunosuppressants such as corticosteroids and/or other synthetic or biological disease-modifying antirheumatic drugs (DMARDs) to achieve control of the inflammation.^2^ Interestingly, the PD-1/PD-L1 axis is also modulated during antiviral responses, both in acute and chronic infections. For example, in acute viral infections like influenza, where antigenic load is considered ‘temporary,’ PD-1 is upregulated on virus-specific CD8 +T cells in order to dampen the subsequent inflammatory response that accompanies viral clearance, and then returns to normal levels as the viral load decreases.^3^ Inhibition of this axis, such as with ICI treatment, could theoretically cause an overactive inflammatory response. Murine studies have shown that acute viral infections in PD-1/PD-L1 knockout-mice can be associated with lethal immunopathology, such as high levels of systemic cytokines, endothelial damage and local tissue damage.^4^ However, if a virus is not cleared and becomes chronic, like hepatitis B, the continued PD-1 expression eventually leads to T-cell exhaustion—a state where T cells lose their cytotoxic effects.^3^ ICI has been proposed to combat chronic viral infections, and although there has been some success in animal models, it has not translated in clinical studies.^5^

It is not known whether patients with cancer treated with ICI are more vulnerable to COVID-19, or more apt to have severe manifestations of the illness. Activated T cells could in theory protect patients from acquiring infection but simultaneously put them at risk for more severe manifestations of COVID-19 due to uncontrolled inflammation. A study from China suggested that patients with cancer, particularly those on targeted treatments or ICI, were more likely to have severe infection,^6^ but in a recent study of patients with lung cancer hospitalized with COVID-19, the anti-PD-1-treated patients did not have more severe disease.^7^ It is also unclear if patients on immunosuppressive medications for treatment of irAE are at greater risk of developing COVID-19 or have worse manifestations, either from immunosuppression itself or the combination of ICI and immunosuppression (a ‘double hit’). In the absence of clear data or guidelines, oncologists and rheumatologists took varied approaches to treatment continuation and discontinuation in the context of the pandemic.

Here, we report the results of a survey of patients enrolled in a large prospective rheumatic irAE registry. Our goal was to assess patient-driven and physician-driven medication changes made early in the pandemic, and to measure the incidence and severity of COVID-19 infections in this unique population.

## Materials and methods

In 2018, we established a prospective registry for patients with rheumatic irAE at the Hospital for Special Surgery in New York City. Patients were referred to our institution from the Tri-State area, often from Memorial Sloan Kettering also in New York City where they received their ICI treatment. On April 16 2020, following the March–April 2020 ‘surge’ of COVID-19 in the New York Tri-State area, we sent a 23-question survey (online supplemental file 1) to the 88 living registry-enrolled patients. A follow-up survey was sent 6 weeks later. Participants who did not fill out the form electronically were contacted by telephone and asked if they wanted to fill out the form verbally. Patients who had their ICI held vs those that continued were compared and a 2-proportion z-test was done to observe for differences. In addition to questions about current cancer status, ICI and immunosuppressant medication use, irAE status, history of COVID-19 symptoms or diagnosed infection, we asked a free-text question, ‘Is there anything else you would like to tell us about how coronavirus has affected you personally?’ We defined definite COVID-19 as having a positive laboratory test, either viral swab (PCR) or serological antibody test, and probable COVID-19 as physician-suspected infection based on symptomology without a positive laboratory test. Symptomatology included one or more of the following symptoms: fever, cough, dyspnea, anosmia or ageusia/dysgeusia.

10.1136/jitc-2020-001550.supp1 Supplementary data

## Results

Of the 88 registry patients, 65 (74%) completed the survey (39 electronically, 26 by telephone). We excluded two respondents who never received ICI, leaving a total of 63 respondents. Survey respondents were similar to the entire registry cohort as shown in table 1. Mean age of survey respondents was 63 years, 59% were female and 70% had been treated with anti-PD-1 or anti-PD-L1 monotherapy. Forty percent of survey respondents were actively being treated with ICI prior to the pandemic (had received a dose within 6 months of the survey being sent out). Twenty-two percent had progression of their cancer on ICI. Eighty percent had an irAE affecting their joints, including one-third with rheumatoid arthritis-like small joint involvement. At the time of the survey, 30% of patients were on no immunosuppressive medications for their irAE, 41% were on corticosteroids and 12% were on a biological agent (TNF or IL-6R inhibitor) for arthritis. None of the patients on immunosuppressive medications had either patient-driven or physician-driven changes made to those medications as a result of the pandemic.

**Table 1:** Patient characteristics

Characteristics	Entire cohort (n=88)	Survey respondents (n=63)
Female, n (%)	52 (59)	37 (59)
Age, mean (SD) years	64.3 (11.8)	63.4 (11.5)
Caucasian, n (%)	75 (85)	56 (89)
BMI, mean (SD)	28.3 (8.4)	28.4 (8.8)
Hypertension, n (%)	34 (39)	23 (37)
Diabetes, n (%)	9 (10)	7 (11)
Pulmonary disorder, n (%)	8 (9)	4 (6.3)
Cancer type, n (%)		
Melanoma	24 (27)	17 (27)
Renal	19 (22)	14 (22)
Urothelial	10 (11)	10 (16)
NSCLC	15 (17)	8 (13)
Other	17 (19)	14 (22)
Active ICI treatment*		
Yes	45 (51)	25 (40)
No	40 (45)	38 (60)
Unknown	3 (3)	0 (0)
Months since last follow-up, med (IQR)	5 (2,9)	3 (1,8)
ICI regimen, n (%)		
Monotherapy PD-1/PD-L1	62 (70)	44 (70)
Combination	26 (30)	19 (30)
Cancer response, n (%)		
CR/PR/stable	67 (82)	49 (78)
Progression	15 (18)	10 (22)
Rheumatologic irAE, n (%)		
Small joint arthritis	32 (36)	22 (35)
Activated OA	15 (17)	9 (14)
PMR	8 (9)	7 (11)
Large joint arthritis	8 (9)	6 (10)
Arthralgia	9 (10)	6 (10)
Sicca syndrome	3 (3)	3 (5)
Myositis	2 (2)	2 (3)
Other†	11 (13)	8 (13)
Arthritis medications‡, n (%)		
No medications	25 (28)	19 (30)
NSAIDs	13 (15)	9 (14)
Steroids	39 (44)	26 (41)
HCQ	11 (15)	6 (10)
MTX	5 (6)	4 (6)
Anti-TNF	5 (6)	5 (6)
Tocilizumab	3 (3)	3 (5)
Arthritis medications changed due to the pandemic	--	0 (0)

*Active ICI treatment=received a dose of ICI within the 6 months preceding the survey.

†Other diagnoses include eosinophilic fasciitis, gout, enthesitis, costochondritis, bursitis.

‡Totals do not add up to 100% given that patients can be on multiple medications at a given time.

BMI, body mass index; CR, complete response; HCQ, hydroxychloroquine; ICI, immune checkpoint inhibitor; irAE, immune-related adverse effect; MTX, methotrexate; NSAIDs, non-steroidal anti-inflammatory drugs; NSCLC, non-small cell lung cancer; OA, osteoarthritis; PMR, polymyalgia rheumatica; PR, partial response; TNF, tumor necrosis factor.

Table 2 highlights the subset of registry patients whose last dose of ICI was within 6 months of the survey. Of the 25 patients, seven (28%) had their ICI held due to concerns regarding the pandemic. There were no significant differences between those who did versus did not hold their ICI, although there was a trend toward more ICI interruption in female patients, those on ICI monotherapy, those with urothelial cancers and those with a good cancer response. None of our registry patients with renal cell cancer (RCC) had their ICI held. None of the patients who had their ICI held had active arthritis or other irAE requiring immunosuppression. 42/63 (67%) patients responded to the second survey including 6 of the 7 (86%) who held their ICI due to pandemic concerns.

**Table 2:** ICI usage patterns in patients on active ICI treatment* (n=25)

Characteristics	Continued their ICI (n=18)	Held their ICI (n=7)
Female, n (%)	8 (44)	5 (71)
Age, mean (SD)	64.4 (12.2)	66.6 (11.3)
Caucasian, n (%)	15 (83)	6 (86)
BMI, mean (SD)	29.5 (12.3)	34.7 (11.2)
Hypertension, n (%)	8 (44)	2 (29)
Diabetes, n (%)	4 (22)	0
Pulmonary disorder, n (%)	0	1 (14)
Cancer type, n (%)		
Melanoma	3 (17)	2 (29)
Renal	4 (22)	0
Urothelial	3 (17)	3 (43)
NSCLC	2 (11)	1 (14)
Other	6 (33)	1 (14)
ICI regimen, n (%)		
Monotherapy	12 (67)	7 (100)
Combination	6 (33)	0 (0)
Cancer response, n (%)		
CR/PR/stable	15 (83)	7 (100)
Progression	3 (17)	0
Immunosuppression†		
No medications	4 (22)	2 (29)
NSAIDs	2 (11)	1 (14)
Steroids	9 (50)	4 (57)
HCQ	2 (11)	1 (14)
MTX	2 (11)	0
Anti-TNF	2 (11)	0
Tocilizumab	1 (6)	0

*Active ICI treatment = has received ICI within 6 months of survey.

†Totals do not add up to 100% given that patients can be on multiple medications at a given time.

BMI, body mass index; CR, complete response; HCQ, hydroxychloroquine; ICI, immune checkpoint inhibitor; irAE, immune-related adverse effect; MTX, methotrexate; NSAIDs, nonsteroidal anti-inflammatory drugs; NSCLC, non-small cell lung cancer; OA, osteoarthritis; PMR, polymyalgia rheumatica; PR, partialresponse; TNF, tumor necrosis factor.

Two have resumed ICI therapy (an 81 male with urothelial cancer with a complete response and a 62 female with non-small cell lung cancer with a complete response), two continue to hold their ICI citing pandemic concerns (a 52 female with melanoma with a complete response and a 65 female with breast cancer with a complete response) and two stopped their therapies due to a femoral fracture or completion of therapy (both female patients with stable urothelial cancer). A total of six patients (10% of survey respondents) had definite or probable COVID-19 (table 3), including 2/18 (11%) that continued ICI through the pandemic and the other 4/38 (11%) who were not actively being treated with ICI.

**Table 3:** Case series of COVID-19 patients (positive and presumed positive)

	Patient 1	Patient 2	Patient 3	Patient 4	Patient 5	Patient 6
Age, sex	79 M	69 F	53 M	41 M	72 M	54 F
Race	Caucasian	Caucasian	African-American	Caucasian	Caucasian	Caucasian
Cancer	Renal cell Carcinoma	Pleomorphic sarcoma	Renal cell Carcinoma	Renal cell Carcinoma	NSCLC	Melanoma
ICI	Monotherapy	Combination	Monotherapy	Combination	Combination	Monotherapy
Last ICI dose	6/4/18	6/1/17	4/1/19	3/6/20	3/18/20	7/26/18
Cancer status	Stable	CR	Stable	PR	Stable	Stable
Arthritis status	Ongoing, stable	Ongoing, stable	Resolved	Ongoing, stable	Ongoing, improving	Resolved
Antirheumatic medications	None	NSAIDs	None	Prednisone 20 mg daily (for ICI-colitis)	Prednisone 7.5 mg daily	None
SARS-CoV-2 Symptoms	Cough, fever, headache, dizziness, weakness, diarrhea, change in taste	Cough, loss of smell and taste	Mental fatigue	Cough, shortness of breath	Loss of smell for 5 days	Asymptomatic
Hospitalized	No	No	No	Yes, for ICI-colitis; never intubated	No	No
Calendar month of illness	April 2020	March 2020	April 2020	March 2020	March 2020	Unknown
State of residence	New York	New York	New York	New York	New Jersey	New York
SARS-CoV-2 testing	Not tested	Not tested	Positive PCR	Positive PCR	Positive PCR	Positive Serology

CR, complete response; F, female; ICI, immune checkpoint inhibitor; M, male; NSAID, nonsteroidal anti-inflammatory drugs; NSCLC, non-small cell lung cancer; PR, partial response.

Box 1 **Sample answers to the survey free text question, grouped by theme**

**Anxiety/fear**

- ‘It made me terrified to leave home’.
- ‘I have concerns being on an immunosuppressive drug during this time’.
- ‘Anxiety that I can’t go outside and have no control of anything’.
- ‘This situation creates a lot of stress. I’m afraid to leave my house’.
- ‘Can’t leave the house…it is frightening’.
- ‘I have fear and anxiety…getting food is not easy’.

**Depression**

- ‘Death of my friends due to the virus’.
- ‘I am lonely’.
- ‘Sad and depressing…can’t visit family’.
- ‘I go day by day. Some days are bad’.

**Frustration being homebound**

- ‘Can’t stand being in the house’.
- ‘I have developed muscle atrophy from not being able to go to gyms. This sucks!’.
- Bored staying at home’.
- ‘Staying home, very isolated’.

**Economic concerns**

- ‘I lost employment’.
- ‘Preoccupied and worried financially. No work, zero income. Future is unknown’.

**Optimism**

- ‘I am doing great’.
- ‘Doing great, going on hikes and yard work in Vermont…no one in sight’.
- ‘Happy that I’m doing well’.
- ‘Doing well, staying indoors’.

One of the six was asymptomatic but reported positive serologies on the second survey, and the remaining five cases had mild symptoms. Mean age among the patients with COVID-19 was 61 (range 41–79), 4/6 were male and 3/6 had RCC, which was disproportionally higher than the overall cohort both for gender and cancer type. Two were still on active ICI (combination therapy in both cases) and both of these patients were also on prednisone (7.5 mg and 20 mg, respectively). The four other patients were no longer on either ICI or immunosuppression.

Thirty-seven patients (59%) responded to the free-text question ‘Is there anything else you would like to tell us about how coronavirus has affected you personally?’ Box 1 provides a representative sample of the responses. Of the 37 respondents, 30 (81%) expressed depression, anxiety/fear, frustration and/or economic hardship while 7 (19%) remained optimistic and positive. When those with a ‘negative’ and ‘positive’ outlook were compared, there were no significant differences in age, gender, ICI usage, immunosuppression usage or cancer status.

## Discussion

In this prospective registry of patients with rheumatic irAE from ICI, 10% had probable or definite COVID-19 during the NY Tri-state pandemic ‘surge’ of March–April 2020. Although the incidence was strikingly high, the severity of disease was low, and all recovered uneventfully, despite some still receiving ICI and low to moderate dose steroids. As in other studies, there was a male predominance among those developing COVID-19. Half of the patients with COVID-19 had RCC, but larger studies are needed to determine whether ICI for the treatment of RCC represents a risk factor for infection, or whether it is a surrogate marker for other characteristics, such as older age. The incidence of COVID-19 was not significantly higher in patients currently on ICI vs off ICI.

Our findings contrast with those of a recent study from New York, the epicenter of the pandemic in the USA from March to April 2020. In that retrospective study of 423 patients with cancer with symptomatic COVID-19, ICI use was found to be an independent predictor of hospitalization and severe respiratory illness, compared with patients with cancer on systemic parenteral chemotherapy, even after adjusting for demographics and comorbidities.^8^ Only 31 patients (7%) in that study were on ICI within the 3-month preceding period, and the patients had a variety of malignancies.

In our study, oncologists held ICI in a quarter of rheumatic irAE patients in the context of the pandemic, particularly in women, those on ICI monotherapy and those who had had a good cancer response. Follow-up survey information sent 6 weeks later revealed that resuming ICI therapy, if held previously, is being done on a case-by-case basis. One-third of survey respondents were on no medications for their rheumatic irAE at the time of the survey, indicating inactive disease. However, 62% were on some form of immunosuppression, from corticosteroids to DMARDs. These patients did not experience higher rates of COVID-19. Furthermore, our patients with COVID-19 all had favorable outcomes, despite some being on moderate doses of steroids. This supports the results of a global registry of rheumatic patients with COVID-19 that found that neither exposure to DMARDs or TNF-inhibitors was associated with an increased risk of hospitalization.^9^ Both Robilotti *et al* and Gianfrancesco *et al* found that corticosteroid use (≥20 mg and ≥10 mg per day equivalent of prednisone, respectively) was associated with increased risk of hospitalization. Our registry rheumatologists made no pre-emptive changes to immunosuppression as a result of the pandemic.

Rheumatic irAE patients voiced concerns about the pandemic, echoed in the general population, of depression, anxiety and economic hardship. However, a small subset remained optimistic. There were no appreciable differences in demographics and characteristics between respondents that answered positively and negatively.

One limitation of our study was the introduction of response bias given that we were unable to reach everyone in our registry (23 patients, 26%) and it is unclear if this was due to reasons related to the pandemic or not. However, we had a high survey response rate of over 70%, which is historically high for survey studies. We were also able to verify information supplied in the survey through medical charts for accuracy. Our survey did not take into account social practices that can limit disease spread such as social distancing, mask-wearing and hand-washing patterns. Our patients, knowing that they are in a vulnerable group, may have practiced these measures quite rigorously which can potentially prevent the infection and/or limit the severity of the virus if they did get it. Our registry also primarily consists of Caucasian patients with few relevant comorbidities such as obesity, diabetes or underlying pulmonary disease. Despite this, our results are notable for a high rate of symptomatic infection (10%) over a short time period, which highlights risk factors our registry patients may have that are not fully elucidated. Furthermore, since not all of our patients were tested for the virus, and some infections are asymptomatic, our results may be an underestimate of the true incidence. It should be noted that these findings are specific to the New York Tri-State area at a particular point in time and thus, cannot be generalized to all patients on ICI with rheumatic irAE, but may be relevant to locales that become ‘hotspots’ over time. A recent study found that the seroprevalence of COVID-19 in the general population of New York City until the month of April was around 20%,^10^ though this also may be an underestimate.

In summary, patients with cancer with rheumatic irAE from ICI may be especially vulnerable to COVID-19 but are not necessarily at risk for severe manifestations of the disease. Studies in larger cohorts will be needed to tease out the combined effect of ICI and immunosuppression on COVID-19 incidence and severity.
