# Supplementary material for: Impact of COVID-19 on patients with rheumatic complications of cancer immunotherapy: results of a registry survey
Source: J Immunother Cancer. 2020 Oct 16;8(2):e001550. doi: 10.1136/jitc-2020-001550 (PMC7569707; doi:10.1136/jitc-2020-001550)
Supplement: Supplementary data [file jitc-2020-001550supp001.pdf]

Questions regarding your cancer immunotherapy:

1. What was the date of your most recent infusion of cancer immunotherapy?
2. Has your immunotherapy been held/stopped? (Y/N)
    a. What was the reason it was stopped/held?
        i. Immunotherapy was not working (cancer progression)
        ii. Concerns about the coronavirus pandemic
        iii. Side effects from immunotherapy
            1. Which side effects____
        iv. Other reason
            1. Other reason_____
3. What was the date of your most recent cancer scan(s)
4. What was the result of your cancer scan? (Complete response, partial response, stable, progression, not sure, other _____)
5. Since your last visit, have you been started on other medications for your cancer? (Y/N) - [if yes, then]
    a. free text name of medication
    b. Date started

Questions regarding any new side effects from immunotherapy

1. Have you had any new side effects from your immunotherapy since your last visit/survey? (Y/N).
    [If yes, then:]
        i. Which problem or part of the body listed below was affected by this side effect?
        ii. For any below marked yes, ask
            1. Were you hospitalized?
            2. May we call you to get more information about this?
    b. Heart
    c. Skin rash
    d. Thyroid
    e. Diabetes
    f. Eye problem
    g. Diarrhea/colitis
    h. Liver test abnormalities
    i. Blood count abnormalities
    j. Neurological problem
    k. Lung problem
    l. Kidney problem
    m. Other problem (specify ___)

The next part of the survey is for patient who have had arthritis/joint pain from their immunotherapy:

2. Do you currently or have you had joint pain as a side effect of immunotherapy (Y/N) – [next sections populate only if yes, If the answer is no then skip to **7 below]

    a. Rate the following as: Without any difficulty, with some difficulty, with much difficulty, unable to do:

        i. Dress yourself, including tying shoelaces and doing buttons?
        ii. Get in and out of bed?
        iii. Lift a full cup or glass to your mouth
        iv. Walk outdoors on flat ground?
        v. Wash and dry your entire body?
        vi. Bend down to pick up clothing from the floor?
        vii. Turn regular faucets on and off?
        viii. Get in and out of a car, bus, train or airplane?
        ix. Walk two miles or three kilometers, if you wish?
        x. Participate in recreational activities, if you wish?

   b. How much pain have you felt due to your arthritis during the last week?
      (0, no pain - 10, extreme pain)
   c. Considering all the ways in which illness and health conditions may affect you at this time, please indicate how you are doing
      (0, very well – 10, very poorly)

3. In terms of your arthritis, which would you consider it to be: [choose one only]
   a. Ongoing, and not controlled by my arthritis treatment
   b. Ongoing at a stable/tolerable level on arthritis treatment
   c. Continuing to improve with arthritis treatment
   d. Resolved, and I'm still on arthritis treatment
   e. Resolved and I'm no longer on arthritis treatment
   f. Resolved and I'm no longer on arthritis treatment, but I have lasting damage to my joints
4. Are you having a flare of your arthritis at this time Y/N [if yes, then]
   a. How long has this flare of your arthritis been going on?
      i. 1-3 days
      ii. 4-7 days
      iii. 8-14 days
      iv. More than 14 days
   b. Please choose a number to indicate the severity of your flare during the last week
      i. 0 (no flare) – 10 (extreme flare)

5. Do you have any tender (painful) joints today? Y/N
   a. Which joints are tender?

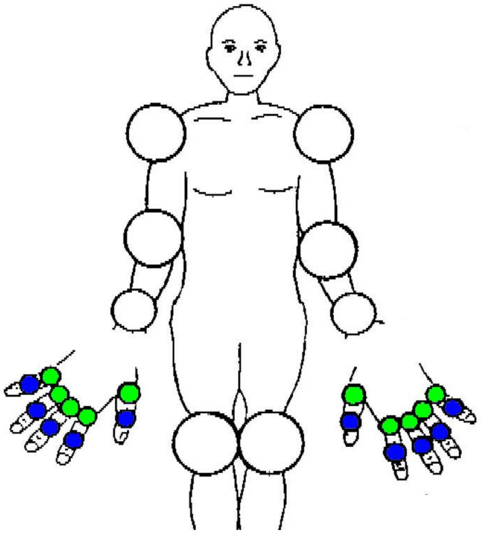

i. Right shoulder
ii. Left shoulder
iii. Right elbow
iv. Left elbow
v. Right wrist
vi. Left wrist
vii. Right thumb blue joint
viii. Right thumb green joint
ix. Right pointer finger blue joint
x. Right pointer finger green joint
xi. Right middle finger blue joint
xii. Right middle finger green joint
xiii. Right ring finger blue joint
xiv. Right ring finger green joint
xv. Right pinky blue joint
xvi. Right pinky green joint
xvii. Left thumb blue joint
xviii. Left thumb green joint
xix. Left pointer finger blue joint
xx. Left pointer finger green joint
xxi. Left middle finger blue joint
xxii. Left middle finger green joint
xxiii. Left ring finger blue joint
xxiv. Left ring finger green joint
xxv. Left pinky blue joint
xxvi. Left pinky green joint
xxvii. Right knee
xxviii. Left knee

6. Do you have any swollen joints today? Y/N
   a. Which swollen joints?

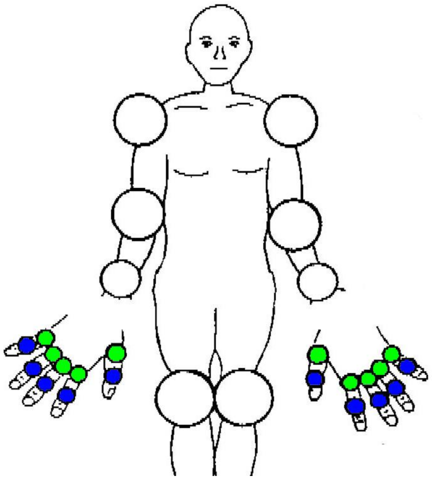

i. Right shoulder
ii. Left shoulder
iii. Right elbow
iv. Left elbow
v. Right wrist
vi. Left wrist
vii. Right thumb blue joint
viii. Right thumb green joint
ix. Right pointer finger blue joint
x. Right pointer finger green joint
xi. Right middle finger blue joint
xii. Right middle finger green joint
xiii. Right ring finger blue joint
xiv. Right ring finger green joint
xv. Right pinky blue joint
xvi. Right pinky green joint
xvii. Left thumb blue joint
xviii. Left thumb green joint
xix. Left pointer finger blue joint
xx. Left pointer finger green joint
xxi. Left middle finger blue joint
xxii. Left middle finger green joint
xxiii. Left ring finger blue joint
xxiv. Left ring finger green joint
xxv. Left pinky blue joint
xxvi. Left pinky green joint

xxvii. Right knee
xxviii. Left knee

****Non-arthritis side effects of immunotherapy**

7. Which non-arthritis side effect of immunotherapy did you see/saw the rheumatologist for?
   a. No non-arthritis side effects
   b. Dry eyes/dry mouth
   c. Muscle aches/weakness
   d. Eosinophilic fasciitis
   e. Gout/pseudogout
   f. Carpal tunnel syndrome
   g. Abnormal blood tests
   h. No rheumatic symptoms; visit was pre-emptive
   i. Other

[(If yes) for any of the above, Is it currently:]

i. Ongoing, and not controlled by treatment prescribed by my rheumatologist
ii. Ongoing at a stable/tolerable level on treatment prescribed by my rheumatologist
iii. Continuing to improve with treatment prescribed by my rheumatologist
iv. Resolved, and I’m still on treatment prescribed by my rheumatologist
v. Resolved, and I’m no longer on treatment prescribed by my rheumatologist
vi. Resolved, and I’m no longer on treatment prescribed by my rheumatologist, but I have lasting damage

8. Which of the following medications are you currently being prescribed for your autoimmune side effects of immunotherapy? [if checked, ask for the date of the last dose]
   a. None
   b. NSAIDS:
      i. Naproxyn/Aleve, ibuprofen/Advil/Motrin, meloxicam/Mobic, diclofenac/Voltaren, other NSAID ___
   c. Prednisone
      i. Current dose
   d. Methylprednisolone
      i. Current dose
   e. Hydroxychloroquine/Plaquenil
      i. Dose (#tablets daily)
   f. Sulfasalazine/Azulfidine
   g. Methotrexate
   h. Leflunomide/Arava
   i. Budesonide
   j. Mycophenolate mofetil/Cellcept
   k. Infliximab/Remicade
   l. Adalimumab/Humira

m. Tocilizumab/Actemra
n. Sarilumab/Kevzara
o. Rituximab
p. Abatacept/Orencia
q. Other _____

9. Have you stopped or changed the dose of any of the medications prescribed by your rheumatologist since your last visit/survey? (Y/N)

[If yes, for any marked above, choose one of these three options]

a. Stopped (date)
b. Reduced dose (date)
c. Reduced frequency (date)

[and ask:]

d. Reason for change?
    i. MD instructed me to due to coronavirus
        1. Rheumatologist
        2. oncologist
    ii. MD instructed me to for other reasons
        1. Rheumatologist
        2. Oncologist
            a. Reason for MD changing:
                i. Side effects
                ii. Improving
                iii. Worsening
                iv. Other
    iii. I decided to on my own (If yes, then ask why)
        1. Medicine wasn't working
        2. Medicine was causing side effect
        3. Coronavirus
            a. I was worried about getting coronavirus
            b. I had symptoms of coronavirus, but was not diagnosed with it
            c. I was diagnosed with coronavirus
        4. Other reason

Now we are going to ask some questions about coronavirus:

10. Have you had close contact with someone with a diagnosis of coronavirus? (Y/N/other/unknown/details)

11. Have you been tested for coronavirus with either a nasal swab or blood test? (Y/N) if yes
    a. date or approximate date
    b. result – positive, negative, unknown

(If no)

c. Have you been diagnosed with coronavirus by a doctor without a test? Y/N

12. Were you hospitalized for coronavirus? Y/N
13. Is there anything else you would like to tell us about how you are doing? (free text)
14. Is there anything else you would like to tell us about how coronavirus has affected you personally? (Free text)
